# La rétinopathie d'irradiation

**DOI:** 10.11604/pamj.2015.22.54.6395

**Published:** 2015-09-18

**Authors:** Fadoua Zahir, Hicham Tahri

**Affiliations:** 1 Service d'Ophtalmologie, CHU Hassan II, Fès, Maroc

**Keywords:** Rétinopathie d′irradiation, pathologie vasculaire rétinienne, maculopathie d′irradiation, radiation retinopathy, retinal vascular disease, radiation maculopathy

## Image en medicine

La rétinopathie d'irradiation est une pathologie vasculaire rétinienne iatrogène, chronique et progressive, secondaire à une radiothérapie de tumeurs intraoculaires ou de tumeurs malignes avoisinantes. Il s'agit d'une microangiopathie rétinienne occlusive retardée et progressive qui regroupe la rétinopathie (ischémique ou proliférante) et la maculopathie (ischémique ou œdémateuse). Une neuropathie seule ou associée à la rétinopathie peut compliquer une exposition à des irradiations. Nous rapportons le cas d'un patient de 45 ans traité par radiothérapie externe pour une tumeur du cavum il y a 4 ans et qui présente actuellement une maculopathie d'irradiation œdémateuse. Les phénomènes exsudatifs secondaires à des vasculopathies sont à l'origine d’œdèmes et d'exsudats, préférentiellement au niveau de la macula. Actuellement, il n'existe aucun traitement établi pour la rétinopathie ou la maculopathie d'irradiation. Le développement de stratégie de prévention reste primordial pour éviter une potentielle cécité.

**Figure 1:**
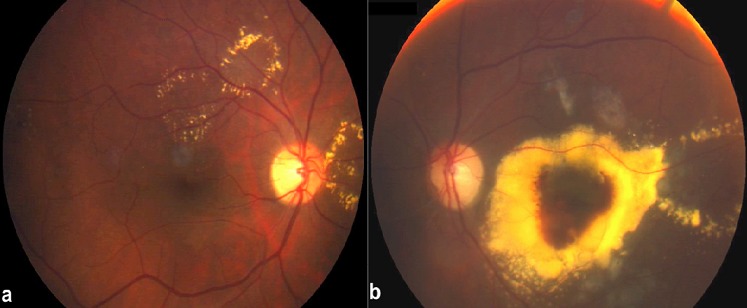
Gros exsudats secs au cours de la maculopathie radique

